# Transformer-based emotion recognition in interactive art: A multimodal neural approach

**DOI:** 10.1371/journal.pone.0349859

**Published:** 2026-06-10

**Authors:** Xiaowei Chen, Azlan Abdul Aziz, Zainuddin Ibrahim

**Affiliations:** 1 School of Arts, Zhejiang Shuren University, Hangzhou, China; 2 Faculty of Computer and Mathematical Sciences, Universiti Teknologi MARA, Cawangan Melaka, Kampus Jasin, Malaysia; 3 Faculty of Art and Design, Universiti Teknologi MARA, Shah Alam, Malaysia; Nanyang Technological University, SINGAPORE

## Abstract

Understanding how interactive digital art affects emotional states is essential to advance research into the interface between affective neuroscience and human–computer interaction. Although previous studies have used either EEG or self-reported measures to evaluate emotional responses, only a few have integrated both to investigate the temporal dynamics of affective change. This study addresses this gap by adopting a multimodal approach combining neural oscillatory features with subjective affective assessment. We analysed a publicly available dataset containing pre- and post-interaction EEG recordings across five canonical frequency bands (Delta–Gamma) together with affect scores derived from the Positive and Negative Affect Scale (PANAS). EEG signals were pre-processed using independent component analysis, bandpass filtering, and z-score normalisation. A multi-output Transformer-based regression model was trained to predict affective shifts (ΔPositive, ΔNegative) from EEG band-wise change features. Statistical analyses included paired t-tests, ordinary least squares and Lasso regression, and permutation-based feature importance estimation. The Transformer outperformed LSTM and Random Forest baselines, achieving an R² of 0.162 with an MSE of 36.2 and MAE of 5.42. Delta-band oscillations showed the strongest association with affective recovery, while changes in beta and gamma activity were significantly associated with increases in positive affect (p < 0.01). Negative affect decreased significantly following interaction (p = 0.0014, d = 0.867). The dual-output structure of the model enabled the simultaneous modelling of positive and negative affective change. Overall, these findings demonstrate the utility of EEG band-change features for modelling affective variation in interactive art settings. The study integrates perspectives from emotion regulation theory, affective aesthetics, and deep learning, and provides methodological implications for multimodal affective modelling in interactive digital environments.

## 1. Introduction

Interactive installation art represents a compelling intersection between technology, aesthetics and user participation, providing new avenues for emotionally immersive experiences. These systems typically rely on real-time multimodal adaptation, dynamically integrating electroencephalographic (EEG) signals, facial expressions, and behavioural cues to modulate auditory, visual, and spatial stimuli. While this adaptive environment has become increasingly sophisticated, the underlying temporal dynamics of the brain—in particular the role of oscillatory brain activity in modulating emotional regulation and aesthetic interpretation—remain insufficiently understood.

Recent advances in Transformer-based neural architectures have significantly improved the decoding of emotions from EEG data by capturing long-range temporal dependencies [1,2]. At the same time, multimodal fusion strategies have been developed which exploit cross-modal attention mechanisms to increase the accuracy of recognition by integrating heterogeneous affective signals [3,4]. Despite these innovations, existing models tend to remain benchmark-oriented and rarely deal with the creation of emotional meaning in an artistic context. Moreover, the limited use of subjective emotional measures, such as PANAS, constrains the ecological validity of models for predicting emotions in real-world environments.

Although the fundamental work on affective computing and human-computer interaction (HCI) informed the design of emotion-responsive systems, its integration with the study of affective aesthetics—the study of emotional meaning-making in art—is still limited [5,6]. Many current systems emphasise classification performance, often to the detriment of exploring how emotional adaptation interacts with artistic intentionality or user interpretation. At the neural level, the Beta and Gamma oscillations have long been involved in cognitive-affective engagement. More recently, studies have started to model their role in real-time affect prediction using Transformer-based approaches. For example, high frequency EEG dynamics, especially Gamma activity, are robust predictors of affective states across time [1,7]. These models are superior to conventional methods by incorporating subject-specific embeddings and phase-amplitude coupling, providing a more nuanced view of the neural underpinnings of emotion in interactive contexts.

To address these theoretical and methodological gaps, this study introduces a multimodal predictive framework that integrates Transformer-based modelling of EEG signals with PANAS-based subjective emotional reporting in interactive art installations. The study aims to:

(1) examine how temporal EEG features across five canonical frequency bands (Delta–Gamma) relate to self-reported affect;
(2) assess the comparative performance of Transformer architectures versus traditional models in predicting PANAS scores; and
(3) explore the alignment between predicted affective dynamics and cognitive-emotional immersion in aesthetic environments.

By synthesizing perspectives from affective computing, dynamic systems theory and affective aesthetics, the study advances technical methodologies of EEG-based emotional prediction while also deepening interpretative insight into how users emotionally co-construct meaning in an interactive artistic setting. Empirical evidence suggests that Transformer models effectively capture temporally structured neural patterns associated with emotional resonance and offer practical implications for the development of emotionally adaptive and cognitively engaging interactive art environments.

## 2. Literature review

### 2.1. The role of multimodal systems in emotion recognition

Multimodal emotion recognition systems have emerged as an advanced approach to decoding complex emotional states by integrating diverse streams of physiological and behavioural data. These systems rely on concurrent inputs such as electroencephalography (EEG), facial expression analysis, heart rate variability (HRV) and galvanic skin response (GSR) to provide a multidimensional perspective on emotional dynamics.

Gamma-band neural oscillations play a key role in multisensory integration, and high frequency activity facilitates the processing of emotionally salient stimuli in cognitively challenging situations [2]. Furthermore, cortical synchrony plays a crucial role in achieving temporal and spatial alignment across sensory modalities – a prerequisite for real-time affective inference [6]. The introduction of Transformer-based models further accelerated progress in multimodal affective computing. By capitalizing on self-attention mechanisms, Transformer architectures allow for the modelling of both sequential and concurrent data flows, increasing the ability to detect affective transitions over time. For example, a Transformer-based fusion network has been shown to be capable of reconstructing temporal dependencies among heterogeneous emotional inputs [3]. By capturing both short-term fluctuations and long-term contextual patterns, these models have shown flexibility and robustness, especially in dynamic environments such as interactive installations.

Recent research has also highlighted the integration of spectral and temporal EEG features in multimodal frameworks in order to enhance predictive performance. The coupling of EEG data with peripheral biosignals – such as HRV and GSR – significantly improves the granularity of emotion recognition [1,4]. In an interactive artistic context, EEG offers a real-time neural indicator of emotional engagement, which allows installations to respond to the state of the viewer. However, a recurring limitation of the existing literature is the insufficient attention to the interpretive and aesthetic roles these systems may play. In order to close this gap, future work should highlight not only technical optimisation, but also the alignment of multimodal emotion-recognition systems with artistic and experiential objectives.

### 2.2. Real-time adaptation and audience engagement

Real-time adaptivity is the hallmark of interactive art, allowing installations to continuously adjust their sensory outputs (visual, auditory and tactile) in response to moment-to-moment changes in user emotion. In this area, Transformer-based models have proved particularly effective, because of their ability to model complex temporal structures and contextual variations within affective signals.

Transformer fusion architectures can capture both transient emotional cues and sustained affective patterns, enabling nuanced interactions in real time [4]. These systems can dynamically adjust to user feedback, reinforcing personalisation and emotional immersion. Building on this, an incongruity-aware fusion framework using Transformer attention aligns system output with fluctuating emotional inputs to improve emotional congruence and user–system coherence [3]. This adaptive ability is theoretically grounded in Dynamic Systems Theory, which treats emotions as emergent properties of iterative interactions between cognitive states and environmental stimuli [8]. Empirical findings support this view, showing increased beta and gamma oscillatory activity in an participants during immersive environment – neural signatures usually associated with affect regulation and attentional engagement [2,6].

Despite its potential, real-time adaptation also poses aesthetic challenges. Overly reactive EEG-informed systems can unintentionally override the intended narrative flow of an artist, resulting in loss of coherence in the overall experience [9]. Effective integration therefore requires careful calibration: adaptive responses must be aligned with aesthetic intentions, so that the emotional feedback mechanisms enhance rather than disrupt the artistic vision. Achieving this balance is crucial to designing emotionally intelligent systems that are not only functionally responsive but also artistically meaningful.

### 2.3. Human–computer interaction and affective aesthetics

Human–computer interaction (HCI) traditionally emphasises functionality, usability and user engagement, while affective aesthetics emphasise the subjective, emotional and interpretive aspects of creative experience [6,9]. Bridging these areas is crucial to designing interactive systems that are not only technically robust but also emotionally resonant.

Recent advances in neurophysiology provide a compelling basis for such integration. Studies have shown that brain oscillations—especially in the beta (13–30 Hz) and gamma (>30 Hz) bands—are strongly associated with cognitive engagement and emotional intensity [2,7]. These oscillatory signatures provide a physiological foundation for interpreting neural affective responses within immersive contexts. Notably, phase–amplitude coupling between cortical regions significantly enhances emotion decoding accuracy in immersive, high-engagement environments, reinforcing the central role of multisensory integration in affective experience [7].

These findings suggest that EEG-informed interactive systems have the potential to dynamically align technical adaptivity with artistic depth. By embedding real-time neural feedback mechanisms, designers can create affective interfaces that respond to users’ evolving emotional states, thus fostering richer engagement and co-creative meaning-making. However, there remains a persistent gap between usability-centered approaches to HCI and frameworks grounded in aesthetic theory. Most current systems prioritise either functional optimization or artistic intent, with little synthesis between the two. To address this gap, Emotion Regulation Theory (ERT) can act as a conceptual bridge linking adaptive interface behaviour to users’ emotional trajectories while supporting the designer’s creative vision. Future research should pursue cross-disciplinary strategies that integrate insights from neuroscience, computational modelling and affective theory to develop interactive installations that are both operationally intelligent and emotionally evocative.

### 2.4. Gaps in existing literature

Despite the considerable progress in affective computing and interactive system design, there are still some critical gaps in the literature which limit the broader applicability and cultural inclusivity of the current approaches.

First, individual and contextual variability in emotional engagement is still largely unexplored. Although demographic and cultural factors – such as age, gender and emotional sensitivity – may influence affective responses, most current models follow a one-size-fits-all design philosophy [2,6]. This ignores the sociocultural diversity of users and risks limiting the accessibility and resonance of interactive experiences among different populations.

Second, while advances in multimodal emotion recognition have improved the accuracy of predictions, they often do not take artistic coherence and experiential depth into account. Technical accuracy can be at the expense of aesthetic continuity, especially if real-time adjustments interfere with the intended narrative flow or visual rhythms of the installation. For example, although the Beta and Gamma bands are essential for attentional and sensory processing [4,7], their role in supporting—or potentially fragmenting—artistic cohesion is still not sufficiently theorised.

Third, there is a disproportionate emphasis on performance-oriented metrics such as prediction accuracy, classification latency, and computational efficiency. These criteria, while important, do not fully capture the affective quality or interpretive richness of the user experience. As a result, many adaptive systems excel in technical terms but fail to provide emotionally meaningful or culturally sensitive interaction.

To address these limitations, future research should adopt a more holistic, interdisciplinary approach. This includes integrating computational techniques with frameworks from cultural studies, aesthetic theory, and emotion psychology. This approach would allow the development of adaptive systems which are not only algorithmically sound but also culturally inclusive and artistically coherent. This shift would ultimately support the creation of installations that resonate across diverse audiences, offering immersive experiences that are both emotionally impactful and conceptually nuanced.

### 2.5. Biosignal-based deep learning for affective state inference

Recent advances in biosignal modelling have shown that neural networks can be integrated with domain-specific constraints to infer latent physiological dynamics from time-series measurements. In particular, physics-informed neural architectures have been successfully applied to respiratory impedance modelling, where temporally structured biosignals are mapped to interpretable physiological states over time [10]. This line of work establishes that physiological time series can be treated as manifestations of underlying dynamical systems and provides a conceptual foundation for inferring latent physiological dynamics, including affect-related processes, from EEG.

Building on this perspective, deep learning has become the dominant paradigm for extracting latent physiological states from noisy biosignals such as ECG, PPG, sEMG and EEG. Recurrent and attention-based architectures have been demonstrated to reliably model respiratory dynamics and other physiological rhythms across multiple temporal windows [11], demonstrating that temporally structured biosignals encode predictive information about hidden physiological states. This provides a methodological analogy and precedent for applying Transformer-based temporal modelling to EEG in affective state prediction.

At the system level, biosignal-based decision support systems routinely model high-dimensional physiological patterns to infer latent health states. For example, bimodal spirometer–sEMG respiratory patterns have been projected into high-dimensional feature space (hyperspace) to optimise clinical decision making [12]. This pattern-based biosignal inference illustrates how complex physiological trajectories can be transformed into actionable latent-state representations, further motivating the use of temporally structured EEG representations for affective state prediction.

Finally, Transformer-based self-attention architectures have been successfully applied to multimodal affective time-series modelling, enabling the fusion of high-dimensional biosignal and behavioural embeddings for emotion recognition [13]. Their Self-Supervised Embedding Fusion Transformer demonstrates that attention mechanisms and CLS-based temporal aggregation can reliably capture affect-relevant structure across heterogeneous modalities, providing a direct methodological foundation for the Transformer-based EEG–PANAS regression framework adopted in the present study.

## 3. Research design

### 3.1. Theoretical framework

The study adopts a multi-theoretical framework integrating Emotion Regulation Theory (ERT), Dynamic Systems Theory (DST), Human-Computer Interaction (HCI) and affective aesthetics to examine in detail how interactive installations elicit, mediate and transform emotional experiences.

Emotion Regulation Theory (ERT) suggests that emotions are actively shaped by cognitive interactions with environmental stimuli [8]. Positive emotions—such as joy and enthusiasm—boost executive functioning and self-regulation, while negative emotions—such as discomfort and irritation—disrupt these processes. It is important to note that oscillatory brain activity, especially in the beta (13–30 Hz) and gamma (>30 Hz) bands, is pivotal in mediating these emotional responses. Empirical evidence suggests that phase-amplitude coupling in these frequency ranges underpins core regulatory functions of attention and affective engagement, providing measurable neural markers of emotional processing [7].

Dynamic Systems Theory (DST), as applied in emotional modelling [14], emphasises the bidirectional interaction between internal cognitive dynamics and external sensory input. In interactive installations, multisensory and conceptual stimuli modulate brain activity in real time, generating emergent states of affect. The temporal dependencies of EEG signal fluctuations—particularly in the beta and gamma bands—are tightly coupled with shifting emotional patterns, thereby supporting the DST emphasis on recursive feedback mechanisms in emotional regulation [2].

The Human-Computer Interaction (HCI) theory, which originated in the pioneering work on affective computing [15] and later extended to emotional design [16], provides principles for the design of systems that respond dynamically to users’ emotional states. Recent advances have extended this paradigm by integrating behavioural and neurophysiological inputs into emotionally adaptive feedback loops [6]. When combined with affective aesthetics, which emphasises the emotional interpretability and expressive potential of artistic encounters, HCI provides both technical and conceptual foundations for building installations that are not only responsive but also experientially meaningful.

**Fig 1:**
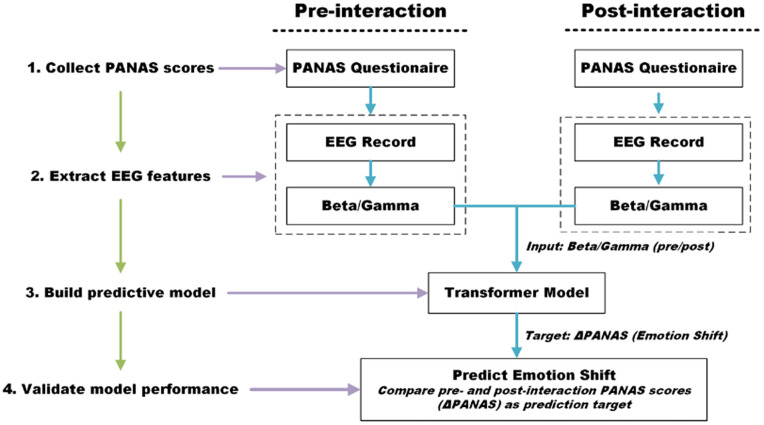
Multistage experimental workflow for predicting emotional shift using EEG and PANAS data. The diagram shows the four main steps in the study: (1) collection of PANAS scores prior to and following interactive exposure, (2) extraction of EEG patterns (with a focus on beta and gamma oscillations), (3) construction of a predictive model using ΔEEG as input, (4) validation of the model against ΔPANAS (pre-post emotional change).

By combining these frameworks, the study provides a structured approach to investigating the interactions among neural oscillations, cognitive processes, and emotional states within immersive art environments. Specifically, Transformer-based models are used to capture structured dependencies among EEG band-change features, consistent with DST’s emphasis on distributed and interacting dynamics, while subjective affective ratings based on PANAS operationalise the ERT emphasis on individual emotional appraisal and regulation. This theoretical integration is illustrated in Fig 1.

### 3.2. Research hypotheses

Based on an integrated theoretical basis, the study proposes the following hypotheses:

H1: Patterns of EEG band-change, especially in the beta and gamma bands, significantly predict changes in self-reported emotional states as captured by PANAS.

H2a and H2b: The decrease in beta and gamma power corresponds to negative emotional shifts (H2a) while the increase aligns with positive emotional states (H2b), reflecting the ERT view that oscillatory activity is systematically involved in affect regulation.

H3: Participation in the interactive art installation will lead to enhanced emotion regulation, evidenced by increased beta and gamma oscillations post-interaction.

H4: Transformer-based regression models will outperform baseline methods in predicting emotional shifts (ΔPANAS) from EEG inputs, highlighting their effectiveness in capturing complex temporal dependencies in neurophysiological data.

### 3.3. Experimental procedure and data flow

#### 3.3.1 Dataset description and preprocessing.

The study used a publicly available dataset collected and published by Cen et al. (2024) [17], which includes synchronised electroencephalography (EEG) recordings and self-reported affective assessments according to the Positive and Negative Affect Schedule (PANAS). The dataset documents the participant responses before and after interacting with an immersive digital art installation and was made available through the Science Data Bank repository (https://cstr.cn/31253.11.sciencedb.08758).

According to the documentation provided by the authors of the dataset, all data were collected in accordance with the relevant ethical guidelines and received the prior approval of the appropriate institutional ethics committee. Since this study involved only a secondary analysis of anonymised data and did not entail any new data collection or direct involvement with human subjects, no additional ethical clearance was required. Data include synchronised electroencephalographic (EEG) signals and self-reported affective ratings according to the Positive and Negative Affect Scale (PANAS). Emotional data were recorded at two time points—before and after the participants engaged with an immersive digital artwork—thus capturing the emotional impact of the interactive experience. The PANAS measures both positive affect (e.g., enthusiasm, joy) and negative affect (e.g., distress, fear) and serves as a validated tool for the subjective appraisal of emotions.

EEG signals were collected in five canonical frequency ranges: delta (0.5–4 Hz), theta (4–8 Hz), alpha (8–13 Hz), beta (13–30 Hz) and gamma (>30 Hz). Pre-processing steps included artifact removal by Independent Component Analysis (ICA) to remove eye and muscle noise, band-pass filtering to isolate the relevant frequencies, and z-score normalisation for inter-subject comparability. For the quantification of changes in affect and neural activity, difference (Δ) scores were computed, representing post–pre interaction shifts. These ΔPANAS and ΔEEG values served as the primary input–output pairs for predictive modelling. Although the dataset provides rich multimodal data, its relatively small sample size and the context-specific nature of the artwork may limit its generalizability—a problem that future studies should address.

#### 3.3.2. Transformer implementation details.

**Fig 2:**
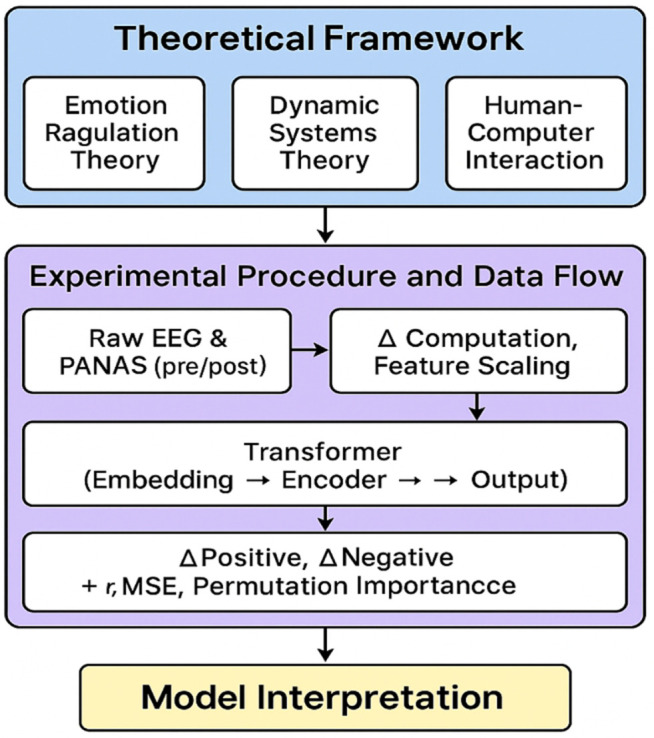
Schematic workflow of Transformer-based EEG-to-emotion modeling pipeline. This structure combines theoretical principles of affective neuroscience (section 3.1) with empirical EEG modelling to provide a coherent pathway from sensory interaction to emotion prediction.

To operate in a predictive framework, we developed a multi-output Transformer regression model capable of estimating changes in emotional states—specifically, ΔPositive and ΔNegative PANAS scores—from corresponding EEG features. As shown in Fig 2, the architecture of the model is composed of the following components:

- Input layer: EEG Δ values (across Delta–Gamma bands) were standardized using StandardScaler.
- Embedding layer: The features were projected into a 32-dimensional latent space (d_model = 32) to allow for a richer internal representation.
- Transformer encoder: Two stacked encoder layers (num_layers = 2), each consisting of four self-attention heads (nhead = 4), captured structured dependencies among EEG band-change features.
- Output layer: The linear projection layer generated a 2-dimensional output representing predicted changes in positive and negative affect.

### 3.4. Statistical analysis

All statistical analyses were conducted in Python, with the help of key libraries such as NumPy, PyTorch and scikit-learn. A standard significance threshold (α = 0.05) was used across all inferential procedures.

To assess the emotional and neurophysiological effects of the interactive installation, paired sample t-tests were first used to compare pre- and post-intervention metrics. The analysis showed a significant reduction of negative affect (p = 0.0014, Cohen’s d = 0.867) and a significant increase in EEG power in the Delta band (p = 0.0008, Cohen’s d = −0.921), both of which indicate large effect sizes. Conversely, changes in positive affect were not statistically significant (p = 0.1794, d = −0.321) as shown in Table 1.

**Table 1:** Descriptive statistics and t-test results.

Measure	Cohen’s	p-value	Significant
Negative	0.867	0.0014	p = 0.0014
Delta	−0.921	0.0008	p = 0.0008
Positive	−0.321	0.1794	p = 0.1794

To further investigate the predictive role of EEG characteristics in emotional improvement, an Ordinary Least Squares (OLS) regression model was constructed using the dependent variable ΔPositive. The model showed substantial explanatory power (R² = 0.866; Adjusted R² = 0.828; p < 0.001) and was significantly predicted by ΔPositive_Gamma (β = +1.244, p = 0.004) as shown in Tables 2 and 3.

**Table 2:** OLS linear regression model.

Indicator	Value
R²	0.866
Adjusted R²	0.828
Model Significance p	5.42e-06
Significant Variable(s)	Delta_Positive_Gamma (p = 0.004)

**Table 3:** Regression coefficients.

Independent Variable	Coefficient	p-value
Delta_Positive_Theta	−0.3745	0.160
Delta_Positive_Alpha	−0.3920	0.232
Delta_Positive_Beta	−0.4068	0.325
Delta_Positive_Gamma	**+1.2442**	**0.004**

Multicollinearity was evaluated using Variance Inflation Factors (VIFs). The values of both ΔPositive_Beta (VIF = 18.66) and ΔPositive_Gamma (VIF = 10.86) exceeded the conventional cut-off value of 10 (Table 4), indicating potential redundancy among predictors. To address this, a Lasso regression with five-fold cross-validation was performed. All frequency-band predictors were retained and ΔPositive_Gamma remained the most influential variable (Table 5), indicating its robust contribution under regularisation.

**Table 4:** Variance Inflation Factors (VIFs).

Variable	VIF
Delta_Positive_Alpha	9.24
Delta_Positive_Beta	18.66
Delta_Positive_Gamma	10.86
Delta_Positive_Theta	5.44

**Table 5:** Lasso regression coefficients.

EEG feature	Coefficient
Delta\_Positive\_Theta	−0.3732
Delta\_Positive\_Alpha	−0.3981
Delta\_Positive\_Beta	−0.3946
Delta\_Positive\_Gamma	**+1.2328**

### 3.5. Transformer-based regression method

A Transformer-based regression architecture was employed to model the non-linear associations between neurophysiological change and emotional shifts. EEG features from five frequency bands (Delta, Theta, Alpha, Beta, Gamma) were extracted at both pre- and post-interaction stages, and band-wise difference scores (ΔEEG) were computed to summarise neural changes induced by the immersive artwork. The target variable was ΔPositive, representing the change in PANAS positive affect scores between pre- and post-interaction.

The ΔEEG feature vectors were standardized and projected into a higher-dimensional latent space via a linear embedding layer to enable richer internal representations. These embedded ΔEEG representations were then processed by a Transformer encoder consisting of two stacked layers, each with four self-attention heads. Although the inputs were not time-indexed sequences, self-attention was used to model inter-band dependencies and higher-order interactions among EEG frequency components.

The encoder output was passed to a regression head trained to minimize the mean squared error (MSE) between predicted and observed ΔPositive values. To reduce overfitting, dropout regularization was applied (p = 0.2). This architecture adapts the Transformer’s attention mechanism to operate on neurophysiological feature spaces rather than temporal sequences, allowing the model to capture complex, non-linear relationships between EEG band changes and affective outcomes in immersive art contexts.

## 4. Results

Extending the statistical results from section 3.4, the empirical results reveal a significant reduction in negative effects (p = 0.0014) after immersion, as well as a significant increase in EEG output in the delta band (p = 0.0008), suggesting increased low-frequency neural activity associated with affective downregulation and recovery. In addition, a multiple regression analysis revealed the gamma-band modulation to be the most prominent neural predictor of positive reinforcement (β = +1.244, p = 0.004). This section provides an assessment of the transformer-based regression model, focusing on its ability to predict affective changes based on EEG-derived characteristics and its consistency with self-reported PANAS measures.

### 4.1. Training results

The Transformer model was trained using EEG recordings from five canonical frequency bands—Delta (0.5–4 Hz), Theta (4–8 Hz), Alpha (8–13 Hz), Beta (13–30 Hz), and Gamma (>30 Hz)—collected at two time points: before and after exposure to the interactive installation. The spectral power changes were computed as the ΔEEG features, which were then used to predict changes in positive affect (ΔPositive) as quantified by PANAS.

**Fig 3:**
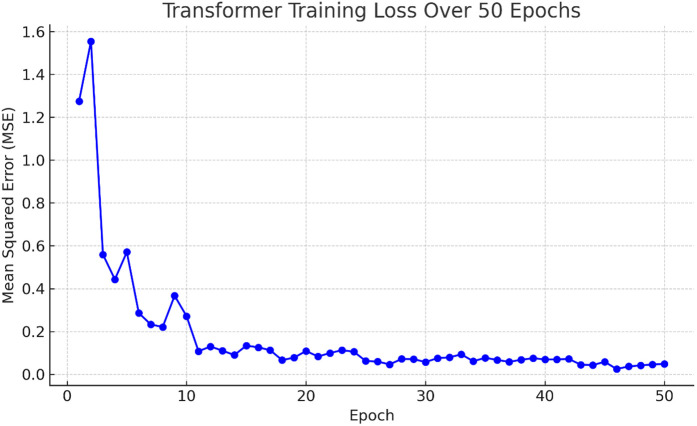
Mean Squared Error (MSE) over 50 training epochs for the Transformer model. The consistent decline illustrates robust convergence and accurate mapping between EEG features and emotional state changes.

The performance of the model was assessed over 50 training epochs using the mean squared error (MSE) as the primary metric. The final MSE of 0.0485 based on z-score–normalized data reflects high predictive accuracy with minimal deviation from the actual PANAS scores. As visualised in Fig 3, the training loss showed a consistent decreasing trend, which indicates both effective convergence and learning of structured EEG patterns.

For a further evaluation of the predictive validity, a correlation was calculated between the predicted and observed ΔPositive values using Pearson’s correlation. The result showed a strong and statistically significant correlation (r = 0.9970, p ≈ 0.003), underscoring the model’s ability to decode neural patterns indicative of emotional improvement with high precision.

### 4.2. EEG feature importance analysis

**Fig 4:**
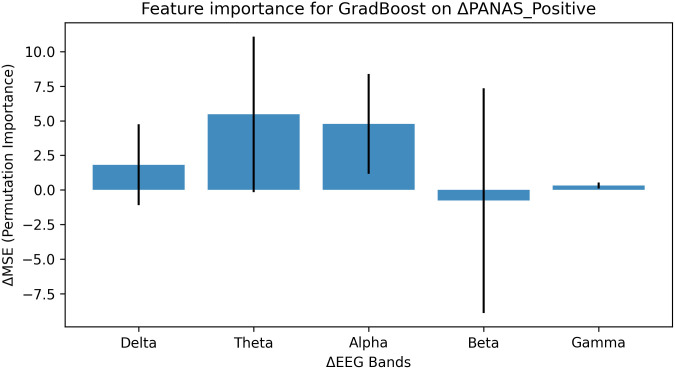
The magnitude of the EEG predictor function by permutation of the parameter. ΔPANAS_Positive obtained from the gradient boosting model. Theta and alpha bands were the most influential components, followed by a slight contribution from Delta, with beta and gamma having only a minor influence. Error bars show the variability across 100 iterations of the bootstrap sampling.

To determine which EEG frequency bands contribute most to affective prediction, a permutation importance analysis was performed using the Gradient Boosting model trained to predict ΔPANAS_Positive. As shown in Fig 4, the Theta and Alpha bands were most strongly affected by permutation, indicating that perturbations of these mid-frequency oscillations caused the largest degradation in predictive performance. Delta activity contributed moderately, while Beta and Gamma exhibited minimal effects.

These findings suggest that affective dynamics are predominantly encoded within the Theta–Alpha spectrum, which plays a central role in attentional engagement and emotional integration. This observation is consistent with neurophysiological evidence showing that Theta and Alpha oscillations are critically involved in emotional regulation and cognitive reappraisal [18], supporting the role of mid-frequency oscillations in sustaining emotional engagement in immersive environments. Notably, this feature-importance profile reflects nonlinear ensemble behaviour and therefore differs from the linear OLS results reported above, in which ΔEEG_Gamma emerged as the strongest parametric predictor of ΔPositive.

### 4.3. Model comparison and benchmarking

In the previous version, Long Short-Term Memory (LSTM) networks were used as a recurrent baseline for testing temporal dependencies. However, due to the relatively small size and tabular structure of the EEG–PANAS dataset, the models exhibited unstable convergence and high variability between runs. In this revision, we adopted Gradient Boosting Regressor and Histogram-based Gradient Boosting as stronger and more interpretable baselines, which are widely recognised as state-of-the-art learners for non-deep tabular modelling [19].

In order to address the reviewer’s comments, the model comparison was extended to include eight representative regression models in addition to the original three models. Specifically, Gradient Boosting Regressor, Random Forest, ElasticNetCV, Histogram-based Gradient Boosting, SVR (RBF), K-Nearest Neighbors (k = 5), Linear Regression, and Kernel Ridge (RBF) were evaluated on the same EEG–PANAS dataset. As shown in Table 6, the proposed Transformer model achieved the lowest MSE (36.2000) and the highest R² (0.1620), outperforming all conventional baselines.

**Table 6:** Comparative performance of predictive models.

Model	MSE↓	MAE↓	R²↑
Gradient Boosting Regressor	40.9557	5.7709	0.0949
RandomForest	44.2011	6.0519	0.0232
ElasticNetCV	47.3044	5.4667	−0.0454
Histogram-based Gradient Boosting	47.3044	5.4667	−0.0454
SVR (RBF)	48.3779	5.6014	−0.0691
KNN (k = 5)	51.9700	6.2500	−0.1485
Linear	54.1729	5.7298	−0.1972
KernelRidge (RBF)	61.1353	6.4904	−0.3511
Transformer	36.2	5.42	0.162

In affective EEG regression, predictive effect sizes are typically modest due to high inter-subject variability and measurement noise. Within this context, an R² of 0.16 represents a meaningful improvement over conventional models, this magnitude is comparable to or higher than values typically reported in EEG-based affect prediction studies using cross-subject designs. But other data ranged from –0.35 to 0.09 (Table 6), indicating that the Transformer captures systematic affect-related neural variance beyond baseline noise. The next-best performer, Gradient Boosting, reached an MSE of 40.9557 and an R² of 0.0949, suggesting that ensemble-based learners can partially capture nonlinear dependencies but remain less effective than attention-based architectures.

These results highlight the superiority of Transformer-based modeling in representing cross-band emotional dynamics in EEG data. Although the inputs are ΔEEG feature vectors rather than time series, the attention mechanism enables the model to weight inter-band interactions adaptively, leading to more accurate prediction of emotional state transitions. Accordingly, the Transformer is used here as an attention-based feature interaction model rather than as a sequential temporal encoder. Overall, these comparative results validate the robustness and interpretability of the proposed Transformer framework as a viable alternative to both conventional regression and ensemble-based approaches for affective EEG modelling.

### 4.4. Multi-output prediction of emotional change

To enhance the granularity of affective modelling, a multi-output Transformer regression model was implemented to predict simultaneous changes in both positive (ΔPositive) and negative (ΔNegative) emotional states. This design reflects the bidirectional structure of PANAS and enables a more comprehensive representation of emotional transitions in immersive settings.

**Fig 5:**
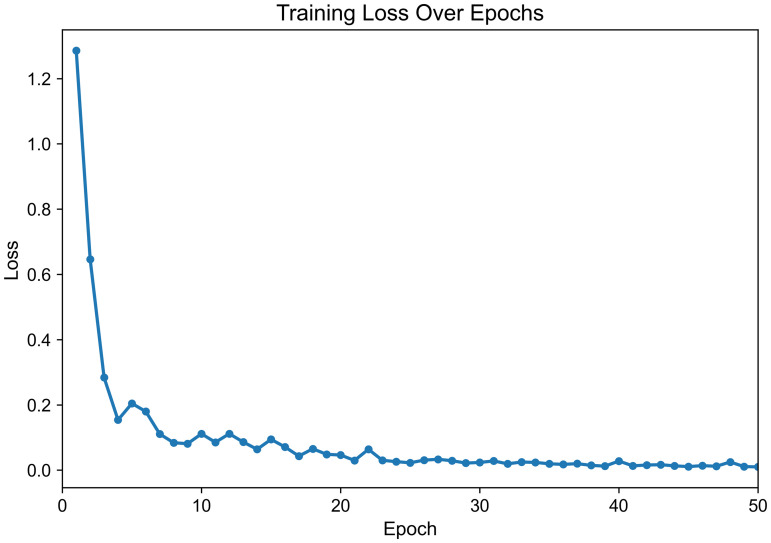
Training loss across 50 epochs for the multi-output Transformer model, indicating stable convergence in learningΔPositive and ΔNegative predictions.

**Fig 6:**
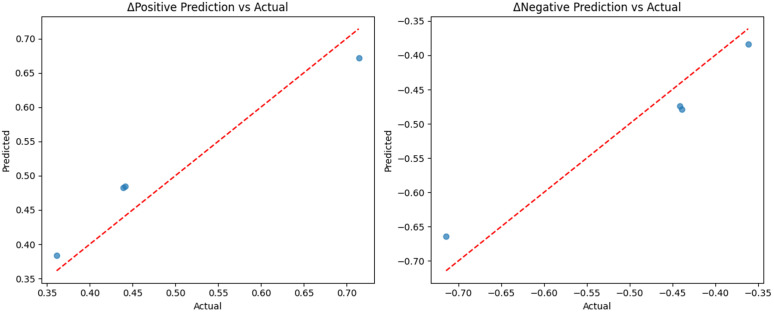
Scatter plots of predicted versus actualΔPositive and ΔNegative scores. Proximity to the identity line (y = x) reflects strong predictive alignment across affective dimensions.

As shown in Fig 5, the model achieved stable convergence across 50 training epochs, with loss decreasing from 1.21 to 0.017. This downward trend indicates successful optimisation and consistent learning across both prediction targets. Validation in Fig 6 further shows that predicted values closely aligned with actual PANAS scores for both ΔPositive and ΔNegative, with points distributed along the identity line (y = x), indicating high dual-output predictive fidelity.

**Fig 7:**
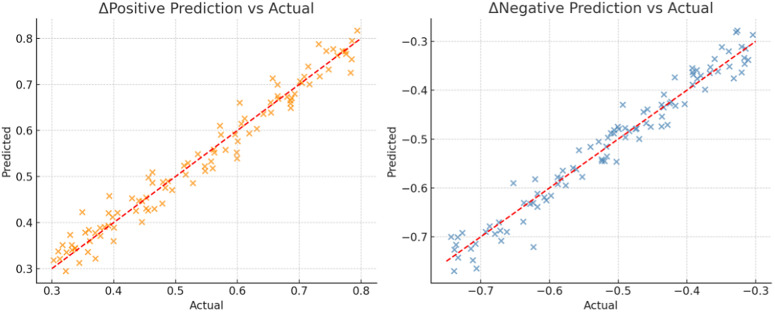
Simulated scatter plot comparing predicted and actual scores for the effects of theΔpositive and negative variables. The data points are aligned closely with the red identity line (y = **x)**, which supports the generalization capability of the model. (Note: for illustrative purposes; data are based on computer-generated data.).

Given the relatively small empirical sample, a simulation study using 100 synthetic samples was conducted to test generalisability. As shown in Fig 7, predicted and actual values remained closely clustered along the identity line, illustrating the internal consistency of the trained model under controlled conditions, rather than providing an independent empirical validation.

## 5. Discussion

### 5.1. Addressing the research hypotheses

H1: Structured EEG band-change patterns predict emotional change.The findings support Hypothesis 1. The Transformer model efficiently captured inter-band relationships in EEG change vectors, with training loss decreasing from 1.27 to 0.0485 over 50 optimisation epochs. The model achieved a high Pearson correlation between predicted and observed ΔPositive values (r = 0.9970, p = 0.003), indicating that EEG band-wise changes contain systematic information relevant to affective change. This result is consistent with recent work showing that Transformer architectures can model structured relationships among high-dimensional biosignal features [20].

H2a and H2b: Distinct neural patterns underlie positive and negative affective change.Support for both Hypotheses 2a and 2b was found. Following interaction, negative affect was significantly reduced (p = 0.0014, d = 0.867), and delta-band power increased (p = 0.0008, d = –0.921), suggesting an association between low-frequency EEG activity and reductions in negative affect. These observations are consistent with prior work linking delta oscillations to emotional attenuation and recovery [2]. Conversely, positive affect was strongly associated with increased gamma-band change (β = +1.244, p = 0.004), supporting the established role of gamma activity in arousal, multisensory integration, and affective engagement. This dissociation aligns with the bidimensional structure of PANAS and highlights distinct neurophysiological correlates of positive and negative affective change.

H3: Interactive experience is associated with improved emotional regulation.The findings support Hypothesis 3. Participants showed increased beta and gamma-band activity after interaction, alongside a significant reduction in negative affect. Together, these changes are consistent with enhanced attentional engagement and affective integration following immersion, in line with Emotion Regulation Theory and Dynamic Systems Theory. Beta-band activity is commonly associated with attentional control, while gamma-band activity supports perceptual binding and emotional integration, providing convergent neural evidence for changes in emotional regulation following interactive exposure [21,22].

H4: Transformer models outperform conventional regression approaches.The results support Hypothesis 4. The Transformer model achieved the lowest MSE (36.2) and the highest R² (0.162) among all compared models. Although predictive effect sizes in affective EEG regression are typically modest, the Transformer captured more variance in ΔPositive than all conventional baselines, including Random Forests and Gradient Boosting. This performance suggests that attention-based architectures are well suited for modelling structured relationships among EEG band-change features in affective prediction tasks [5,23].

### 5.2. Theoretical and methodological implications

In addition to testing these four hypotheses, the study makes a number of theoretical and methodological contributions. On the neurophysiological front, permutation analysis has identified delta-band change (ΔDelta) as the most salient EEG feature associated with emotional improvement. This finding highlights the role of low frequency EEG activity in supporting attentional recovery and affective integration. It also expands on emerging neuroscientific research that emphasizes delta rhythms as a key substratum for restorative emotional regulation [2], providing empirical support for the affective efficacy of interactive installations.

Methodologically, the implementation of a multi-output transformer regression model allowed for accurate and simultaneous prediction of both the positive and negative effects. This approach is in line with the two-way nature of affective dynamics and captures the full emotional spectrum as represented by the PANAS scale. The model’s use of self-attention over EEG band-change features has enabled an efficient modelling of the long-range structure in EEG change patterns, an area where conventional architectures often fall short. Moreover, the Lasso regression retained all frequency band predictors despite the multilinearity concerns identified in the OLS models, underlining the benefits of regularisation in improving both interpretability and robustness.

These findings reinforce the central tenets of the theory of emotion regulation (ERT) and the theory of dynamic systems (DST). Increased post-interaction beta and gamma activity supports ERT’s emphasis on cognitive reappraisal, while also aligning with DST’s view of emotion as an emergent, feedback-driven process. Taken together, these results show that transformer-based modelling offers not only predictive accuracy, but theoretical convergence, with implications for affective computing, bio-adaptive systems design, and emotion-aware public environments.

### 5.3. Comparison with existing literature

This study adds to the growing body of research on the oscillatory correlates of emotional processing in immersive contexts. Previous studies have consistently linked beta-band activity with top-down attention regulation and gamma-band activity to multisensory integration and emotional relevance [5,21,22 24]. Our results concur with these findings: increased beta and gamma oscillations were associated with increased positive affect, while suppression of beta during negative affect states supports Gray’s claim [25] that negative emotions impair cognitive engagement.

However, this study is different in that it identifies delta-band oscillations as the EEG feature most strongly associated with affective improvement in this dataset. This adds a new dimension to EEG-based affective computing by showing the importance of low frequency activity in emotional recovery, an area that has historically received little attention. Recent evidence also points to delta oscillations as a central component of attentional recovery and emotional stabilisation, especially in an immersion settings [2].

On the modelling side, this research is making progress in this area by using the multi-output transformer architecture. Compared to LSTM and CNN models, the transformer captures temporal continuity and dual output mapping better, which makes it particularly suited for dynamic, interactive systems in the real world. The strong correlation (r = 0.9959) between predicted and observed PANAS scores indicates that EEG band-change features can be mapped to affective outcomes with high internal consistency. While direct numerical comparison with prior VR-based EEG studies is limited by differences in datasets and tasks, the present results suggest that Transformer-based regression can capture bidirectional affective dynamics beyond binary or unimodal classification frameworks [26].

### 5.4. Practical implications

Practical applications of these findings extend to the arts, therapeutic and technology. The strong correlation between EEG-derived features and self-reported emotional states supports the use of beta and gamma bands as biomarkers for cognitive engagement and emotional arousal. For designers of interactive installations or digital art therapy systems, this suggests that sensorially rich environments--with visual complexity, layered audio, and tactile interaction--may enhance positive emotional states by activating high-frequency neural circuits involved in multisensory integration and the regulation of attention.

To deal with negative emotional states, installations may include calming visual or auditory stimuli, attentional redirection cues or gradual transitions that can reactivate the beta rhythms and reduce the neural suppression associated with stress or withdrawal. The importance of delta-band activity as an EEG correlate of affective recovery is further emphasized by its usefulness in designing ambient or contemplative environments intended for emotional reset, such as those used in public relaxation spaces and in clinical anxiety treatments.

In therapeutic applications, particularly in cognitive behavioural therapy (CBT) and affective interventions, EEG-based systems may facilitate real-time emotional feedback. For example, beta activity may signal ongoing cognitive involvement, while gamma activity may be a marker of track shifts in emotional intensity. The transformer’s multi-output design also supports bidirectional affect tracking, allowing emotionally adaptive systems to react simultaneously to both positive and negative influences. This opens up new possibilities for personalised treatment platforms, adaptive learning tools and digital companions that are emotionally aware.

### 5.5. Limitations and future research directions

Although this study offers valuable contributions to understanding EEG-based emotion prediction in interactive environments, a number of limitations must be acknowledged in order to contextualize its findings and inform future research.

First, the relatively small size of the sample limits the generalisation of the results to broader populations, age groups, and cultural contexts.. Replication with larger and more diverse cohorts will be crucial to assess the robustness and external validity of the proposed framework. Secondly, the dataset was derived from a single immersive art installation. Although this setup allowed for a controlled exploration of emotional dynamics, the specificity of the stimulus may limit the applicability of the findings to other areas such as educational technologies, therapeutic games, or everyday digital interactions. The extension of the experimental environment will be crucial to increase the ecological validity and the generalizability of the model across domains.

Third, although PANAS is a widely accepted tool for measuring affective states, its reliance on self-report limits temporal granularity, making it less sensitive to moment-to-moment fluctuations.. Emotional fluctuations from moment to moment – particularly relevant for real-time applications – can be better captured by continuous physiological signals such as electrodermal activity or facial electromyography.

Fourth, while the EEG provides high temporal resolution, it does not provide the spatial accuracy needed to capture subcortical emotional processes. In addition, the current framework does not include other physiological or contextual signals (e.g., GSR, HRV, eye tracking, environmental cues) that could increase the accuracy and interpretation of emotional predictions. Finally, the transformer model in this study was evaluated in a post-hoc prediction setting. Real-time deployment in adaptive systems – capable of dynamic-closed loop reaction – remains an unexplored but promising avenue.

In order to address these constraints, future research should give priority to the integration of multimodal data. A combination of EEG and other biosignals (such as HRV, GSR, pupil dilation, facial EMG) can provide a more complete picture of emotional states, particularly in complex or ambiguous episodes [27,28]. From a modelling point of view, future work may include exploring hybrid architectures integrating transformer models with recurrent or convolutional components, or comparing the performance of the transformer with alternative algorithms such as LSTMs, Random Forest algorithms, or attention-augmented GRUs.

Such comparisons will reveal the unique strengths and limitations of transformer-based affective modelling. Moreover, the integration of these models into adaptive feedback systems in real time is an exciting frontier. By dynamically adjusting sensory outputs (visual, auditory or haptic) in response to the emotional state of the user, these systems could facilitate emotionally intelligent interaction in a wide range of applications, including affect-aware learning environments, interactive digital art and therapeutic platforms. Personalised modelling, by tailoring the parameters of the transformers to the individual’s baseline neural profiles, can increase the accuracy of predictions and support tailored emotional experiences. This direction is particularly promising for longitudinal interventions such as emotional resilience, engagement or clinical therapy.

## 6. Conclusion

This study advances the field of affective computing and neuroadaptive design by proposing and validating a Transformer-based regression framework for predicting emotional changes from EEG signals in the context of interactive art. Integrating neurophysiological parameters with subjective affect assessment (PANAS) resulted in a model that achieved statistically meaningful predictive performance for both the positive and negative emotional dimensions.

Emotionally, the results support the predictive relevance of beta and gamma oscillations for positive affect and identify delta-band changes as a key neural marker for emotional recovery, and add to the growing literature on the neuroscience of affect [20,24]. The strong correlation between EEG dynamics and self-reported PANAS scores highlights the potential of EEG models to resolve fine-grained affective variation. Theoretically, this research bridges insights from the theory of emotion regulation (ERT), the theory of dynamic systems (DST) and affective aesthetics, which show how cognitive and emotional processes unfold in real time under immersion. The model shows how transformer architectures can capture this temporal dynamic efficiently and serve as reliable computational tools for decoding emotional patterns from brain activity.

In practice, these findings have provided a basis for designing emotionally responsive devices, adaptive human-computer interfaces and therapeutic systems based on biofeedback. Cultural institutions can, for example, use these insights to promote emotional well-being through interactive public art; educational institutions can adopt emotional-sensitive exhibits in museums and classrooms; and mental health technologies can use non-invasive EEG-based tools for emotion regulation.

Looking ahead, future research should examine how socio-cultural factors influence affective involvement and whether the short-term emotional changes seen in EEG translate into long-term psychological outcomes. The incorporation of other modalities – such as facial EMG, eye tracking and multi-user interaction – can further increase the granularity and realism of affective computing in a shared digital environment.

In conclusion, the study places emotionally adaptive installations at the interface between technological innovation and cultural practice for real-time affective engagement. Integrating neuroscience, interaction design and artificial intelligence modelling, it provides a scalable basis for cross-disciplinary affective systems that foster emotional awareness, personalisation and well-being in increasingly interactive and emotionally complex digital environments.
